# Mycobiome Sequencing and Analysis Applied to Fungal Community Profiling of the Lower Respiratory Tract During Fungal Pathogenesis

**DOI:** 10.3389/fmicb.2019.00512

**Published:** 2019-03-15

**Authors:** Lisa R. McTaggart, Julia K. Copeland, Anuradha Surendra, Pauline W. Wang, Shahid Husain, Bryan Coburn, David S. Guttman, Julianne V. Kus

**Affiliations:** ^1^Public Health Ontario, Toronto, ON, Canada; ^2^Centre for the Analysis of Genome Evolution and Function, University of Toronto, Toronto, ON, Canada; ^3^National Research Council of Canada, Ottawa, ON, Canada; ^4^Department of Cell and Systems Biology, University of Toronto, Toronto, ON, Canada; ^5^Division of Infectious Diseases, Toronto General Hospital Research Institute, University Health Network, Toronto, ON, Canada; ^6^Department of Medicine, University of Toronto, Toronto, ON, Canada; ^7^Department of Laboratory Medicine and Pathobiology, University of Toronto, Toronto, ON, Canada

**Keywords:** mycobiome, internal transcribed spacer, mock community, respiratory tract, *Blastomyces*

## Abstract

Invasive fungal infections are an increasingly important cause of human morbidity and mortality. We generated a next-generation sequencing (NGS)-based method designed to detect a wide range of fungi and applied it to analysis of the fungal microbiome (mycobiome) of the lung during fungal infection. Internal transcribed spacer 1 (ITS1) amplicon sequencing and a custom analysis pipeline detected 96% of species from three mock communities comprised of potential fungal lung pathogens with good recapitulation of the expected species distributions (Pearson correlation coefficients *r* = 0.63, *p* = 0.004; *r* = 0.71, *p* < 0.001; *r* = 0.62, *p* = 0.002). We used this pipeline to analyze mycobiomes of bronchoalveolar lavage (BAL) specimens classified as culture-negative (*n* = 50) or culture-positive (*n* = 39) for *Blastomyces dermatitidis/gilchristii*, the causative agent of North America blastomycosis. Detected in 91.4% of the culture-positive samples, *Blastomyces* dominated (>50% relative abundance) the mycobiome in 68.6% of these culture-positive samples but was absent in culture-negative samples. To overcome any bias in relative abundance due to between-sample variation in fungal biomass, an abundance-weighting calculation was used to normalize the data by accounting for sample-specific PCR cycle number and PCR product concentration data utilized during sample preparation. After normalization, there was a statistically significant greater overall abundance of ITS1 amplicon in the *Blastomyces*-culture-positive samples versus culture-negative samples. Moreover, the normalization revealed a greater biomass of yeast and environmental fungi in several *Blastomyces*-culture-positive samples than in the culture-negative samples. Successful detection of *Coccidioides*, *Scedosporium*, *Phaeoacremonium*, and *Aspergillus* in 6 additional culture-positive BALs by ITS1 amplicon sequencing demonstrates the ability of this method to detect a broad range of fungi from clinical specimens, suggesting that it may be a potentially useful adjunct to traditional fungal microbiological testing for the diagnosis of respiratory mycoses.

## Introduction

Invasive fungal infections are estimated to kill 1.5 million people per year worldwide (Brown et al., 2012). Although they can affect any organ system, the respiratory tract is a prominent portal of access for filamentous fungi to enter the body and establish infection (Brown et al., 2012). Analysis of the fungal microbiome (the mycobiome) has yielded significant insight into the role of fungal communities in human health and disease (Cui et al., 2013) and the characterization of the fungal ecology of various environments (Bik et al., 2012). Characterization of the mycobiome of the lung in chronic respiratory diseases such as cystic fibrosis, lung transplantation, bronchiectasis, asthma, allergic fungal disease, and chronic obstructive pulmonary disease (COPD) or in those with immunosuppression due to HIV reveal substantial quantitative and qualitative variability between individuals and within individuals over time, making it difficult to determine if these opportunistic fungi are involved in disease processes or if they are colonizing or transient residents. *Candida* and *Aspergillus* are commonly detected in the respiratory tract in addition to *Penicillium*, *Scedosporium*, *Saccharomyces*, *Ceriporia*, and *Pneumocystis* (the latter associated with COPD and HIV) (Bousbia et al., 2012; Charlson et al., 2012; Delhaes et al., 2012; van Woerden et al., 2013; Bittinger et al., 2014; Willger et al., 2014; Cui et al., 2015; Kim et al., 2015; Kramer et al., 2015; Krause et al., 2016, 2017; Tipton et al., 2017; Botterel et al., 2018; Fraczek et al., 2018).

North American blastomycosis, is caused by *Blastomyces dermatitidis/gilchristii* which are endemic to regions of Canada and the United States along the Great Lakes, and Saint Lawrence, Mississippi, and Ohio River valleys (Saccente and Woods, 2010). Blastomycosis is a serious fungal infection in both immunocompromised and immunocompetent hosts, presenting as an acute or chronic pulmonary infection with possible progression to serious invasive disease following subsequent dissemination to other organ systems (e.g., skin, bone, genitourinary, CNS) (Pfaller and Diekema, 2010). Mortality is estimated at 6–8% (Chu et al., 2006; Pfaller and Diekema, 2010). Symptoms are not specific and mycoses such as blastomycosis are often omitted from the differential diagnosis, thus, diagnosis relies heavily on microbiology laboratory analysis. Microscopic detection from respiratory samples is rapid but suffers from low sensitivity, while the diagnostic gold standard of positive culture is slow, requiring 2–6 weeks (Saccente and Woods, 2010; Arvanitis et al., 2014). These traditional morphologically based methods of detection of blastomycosis and other pulmonary mycoses require extensive experience on the part of the laboratorian and can still be somewhat subjective. We posit that mycobiome analysis could potentially be a useful adjunct to support traditional fungal microbiological testing in the clinical laboratory (Miller et al., 2013).

Our goals were (1) to develop a robust, accurate NGS-based amplicon sequencing strategy validated against mock communities of a wide range of potential fungal lung pathogens including both acute and chronic airway fungi and (2) to deploy this tool to examine the mycobiome of the bronchoalveolar lavage (BAL) specimens from the lower respiratory tract with known fungal culture status. We sought to investigate the use of this method to describe the lung mycobiome in patients being investigated for fungal respiratory infections as a potential tool to aid in identifying fungi implicated in disease. We specifically examined specimens known to be culture positive or negative for *Blastomyces* species. With other potential fungal pathogens, such as *Aspergillus*, detection of the pathogen alone is generally insufficient to make a diagnosis (host factors, clinical, laboratory and radiographic criteria are integrated to make a diagnosis). However, detection of *Blastomyces dermatitidis/gilchristii* is diagnostic, thus making it an ideal organism to begin exploring mycobiome analysis as a potential adjunct to traditional clinical diagnostic methods.

## Materials and Methods

### Mock Community Preparation

Three mock communities (MC1, MC2, and MC3) were prepared from subsets (20, 20, and 21 strains, respectively) of 53 species of fungi from our collection with an emphasis on lung pathogens (Supplementary Table 1). Individual strains were identified by morphologic examination and Sanger sequence analysis of the ITS1-5.8S-ITS2 region. DNA was isolated from each strain using the DNeasy PowerSoil kit (Qiagen, Germantown, United States) according to the manufacturer’s instructions. Following PCR amplification and sequencing by ITS5 (forward) and ITS4 (reverse) primers (Supplementary Table 2), identification was provided by the top hit (≥99% identity) of a BLASTn search against the NCBI refseq_genomic database (O’Leary et al., 2016). The DNA was quantified using the Qubit^®^ dsDNA BR assay kit (ThermoFisher Scientific) and pooled to so that the mock communities contained 50 ng of DNA of each species (total DNA concentration) (Supplementary Table 1). MC1 was also serially diluted to 1:10, 1:100, and 1:1000.

### Clinical Specimens

The BAL specimens examined in this study were received by the Mycology section, Public Health Ontario between 2011 and 2015. Patient information associated with these specimens was not available. Upon receipt, the specimens were examined microscopically with the aid of calcofluor white for the presence of fungal elements including filaments, yeast and the distinctive large broad-based budding yeast (*Blastomyces*), and cultured on IMA and sheep blood-Cycloheximide (0.01%)-Chlorhexidine (50 μg/ml)-gentamicin (20 μg/ml) (BCCG) media for 4 weeks at 28°C with regular examination for growth. Concurrently, a 1 ml aliquot was archived at −80°C. For the current study, we selected 51 fungal-culture-positive specimens (*Blastomyces dermatitidis/gilchristii n* = 39; *Histoplasma capsulatum n* = 6, *Coccidioides immitis*/*posadasii n* = 3; *Scedosporium apiospermum n* = 1; *Phaeoacremonium* sp. *n* = 1; *Phaeoacremonium* sp. and *Aspergillus fumigatus n* = 1) and 50 fungal-culture-negative specimens (Supplementary Table 3). Specimens were retrieved from −80°C for DNA isolation using the DNeasy PowerSoil kit (Qiagen).

### PCR and Next-Generation Sequencing

Amplicon-based sequencing libraries were generated using the following primer combinations targeting the ITS1, ITS2, or the ITS1-5.8S-ITS2 regions: ITSF/ITSR, ITS3/ITS4, and ITSF/ITS4 (Supplementary Table 2) as previously described (Kim et al., 2015) with minor modifications. Efficiency of primer binding to each of the mock community species was determined by calculating primer scores (Supplementary Table 1) using the primer analysis modules (defaults settings) of Primer Prospector (Walters et al., 2011) and 18S-28S sequences generated with ITS-BMB-CR (forward) and ITS-LR1 (reverse) primers (Supplementary Table 2). PCR of mock communities and BAL specimens utilized the KAPA2G Robust HotStart Ready Mix (Sigma-Aldrich, St. Louis, MO, United States) according to the manufacturer’s instructions and PCR cycling conditions of 25, 28, 30, 33, or 35 cycles. Since the fungal presence in some samples was low, the goal was to reduce amplification bias and thus artificial skewing of the mycobiome profile by using the least number of PCR cycles to generate visible ITS products (∼200–700 bp owing to the size variability of the ITS1 products) on an agarose gel. A threshold of detection of 35 cycles of amplification was set, above which there was a significant enrichment in the presence of contaminants. Of 101 samples, 47/51 (92.2%) culture-positive and 22/50 (44%) culture-negative samples had positive amplification. All PCR reactions were performed in triplicate which were pooled for subsequent steps; samples that failed to produce PCR products at 35 cycles were excluded from further analysis. Non-template DNA isolation controls (NTC) performed by passing PCR-grade water through the DNA isolation kit and PCR negative controls were included as well.

Pooled amplicons were prepared for NGS using the Nextera XT DNA Library Prep kit and Index kit (Illumina, San Diego, CA, United States) as per the manufacturer’s protocol. Barcoded samples were quantified using the Qubit^®^ HS dsDNA assay kit and multiplexed into a single library normalized to 5 ng per sample. Size selection of a ∼350–450 bp band was performed by gel purification and sequenced on the Illumina^®^ MiSeq^®^ with the v2 sequencing kit with 150X2 PE reads. Sequences were deposited in the NCBI Sequence Read Archive under accession number PRJNA516455.

### Mycobiome Analysis

Demultiplexing and adaptor trimming was performed on the MiSeq. Subsequently, the sequences were merged and filtered to discard reads with >0.5 expected errors in USEARCH v9.2.64 (Edgar, 2013). Following the UPARSE pipeline available in USEARCH v9.2.64, sequences were then de-replicated, filtered to remove chimeras, and *de novo* OTUs (operational taxonomic units) picked at 97% sequence identity. OTU taxonomy was identified using a BLASTn database containing a subset of fungal ITS sequences annotated as such in the International Nucleotide Sequence Database and a modified version of FHiTINGS (Altschul et al., 1990; Nilsson et al., 2009; Dannemiller et al., 2014). Based on the top 10 BLASTn results (percent identity ≥0.95, query coverage ≥0.9, *e*-value < 0.000001), taxonomy was assigned to each OTU using a hit abundance cut-off of ≥0.8. Thus, the taxonomy was reported if ≥8 of 10 hits were identical; otherwise the taxonomy was reported as “Ambiguous.” Since the FHiTINGS database contains only fungi, all OTUs where taxonomy was not assigned were queried against the NCBI BLAST nr database. While the mock communities did not contain any non-fungal OTUs, 685 of 1213 OTUs (57%) generated with the BAL samples were non-fungal (107 Bacteria, 84 Plantae, 445 Animalia, 49 Viruses). These OTUs were excluded from further analysis. The proportion of fungal reads recovered from each sample is listed in Supplementary Table 3. Many ambiguities at the genus and species level were clarified by manually reviewing the BLAST results and taxonomies to standardize the anamorph/teleomorph fungal species designations. Regarding the *Ajellomycetaceae*, taxonomies were normalized to *Blastomyces dermatitidis*/*gilchristii*, *Blastomyces percursus*, and *Histoplasma capsulatum* to align with recent taxonomic reassignments (Jiang et al., 2018). *Aspergillus* and *Penicillium* taxa were summarized to section level rather than species level as is appropriate for the level of taxonomic discrimination provided by the ITS1 region (Skouboe et al., 1999; Geiser et al., 2007; Schoch et al., 2012). OTUs with <0.1% relative abundance were filtered from each sample. Based on rarefaction and alpha diversity calculations in Qiime v 1.9.1 (Caporaso et al., 2010) the mock community samples and BAL samples were rarefied to 3000 sequences and 5000 sequences, respectively, with the goal of rarefying to the highest depth possible while minimizing the number of samples excluded (Weiss et al., 2017). Seven culture-negative samples were excluded because they had <5000 fungal reads (range 141–3639, median 1473) due to a low fungal read proportion (range 2.2–52%, median 29%).

Genus-level taxon summaries, alpha diversity analysis, beta diversity analysis with PCoA and UPGMA cluster analysis were performed in QIIME. Additional statistical analyses were performed in R v3.4.2 with packages vegan v2.4-4 (Crist et al., 2003; Oksanen et al., 2017) and pairwiseAdonis v0.0.1 (Martinez-Arbizu, 2017) used for PERMANOVA, ggplot2 v2.2.1 (Wickham, 2009) to construct bar charts and PMCMR v4.1 (Pohlert, 2014) to calculate the Kruskal–Wallis rank sum test with pairwise comparisons using the Tukey and Kramer (Nemenyi) test with a Tukey-distribution approximation for independent samples. For the BAL samples, total and taxon normalized ITS1 abundances (Bittinger et al., 2014) were calculated as:

$$\begin{array}{l} \mathrm{Normalized abundance}=\mathrm{relative abundance}\times \left(\frac{\mathrm{total fungal reads}}{\mathrm{total reads}}\right)\times \\ \left(\mathrm{PCR product concentration}\left(\frac{\mathrm{ng}}{\mu l}\right)\right)\times \left(\frac{1}{2^{\mathrm{No}\mathrm{.PCRcycles}}}\right)\\ \times \left(\frac{25\mu l}{\mathrm{volume}\;(\mu l)\;\mathrm{DNA input}}\right)\times \left(10^{9}\right) \end{array}$$

This value is expressed as arbitrary units (a.u.).

This research project was reviewed and approved by the University Health Network’s Research Ethics Board, and the Public Health Ontario Ethics Review Board. Participant consent was not required for this project, as it involved the analysis of non-identifiable banked clinical specimens. A Privacy Impact Assessment at Public Health Ontario was also completed.

## Results

### Comparison of Various ITS Regions for Optimal Mycobiome Detection and Reassembly

**FIGURE 1:**
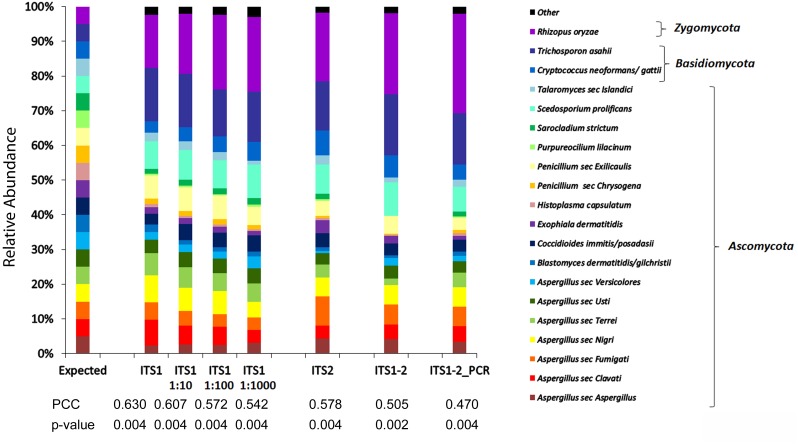
Comparison of mycobiome profiles fungal species of MC1 generated at different concentrations or using different targets. Histogram of relative abundances of each fungal species detected using ITS1 and serial dilutions (1:10, 1:100, 1:1000) of DNA or using alternate targets ITS1, ITS2, ITS1-2, or a mixture of equal concentrations of ITS1 and ITS2 PCR products (ITS1-2 PCR). Pearson Correlation Coefficients (PCCs) suggest that the ITS1 relative abundance profile more closely approximates the expected profile compared to ITS2, ITS1-2, or ITS1-2_PCR. The taxon relative abundance remains relatively consistent over a broad range of input concentrations.

The nuclear ribosomal internal transcribed spacer (ITS) region is the accepted universal DNA barcode marker for fungi (Schoch et al., 2012). We first compared the precision of the mycobiome analysis using various ITS regions (ITS1, ITS2, ITS1-5.8S-ITS2, and equal mixtures of ITS1 and ITS2 PCR products) for the detection of various fungi, including the pathogen of interest (*Blastomyces dermatitidis*/*gilchristii*), in a mock community of equal mixtures of DNA from a total of 20 fungal species (MC1, Supplementary Table 1). The mycobiome profile generated with primers targeting ITS1 most closely approximated the expected taxon relative abundance profile with higher Pearson correlation coefficients (PCCs) than any of the other target regions (Figure 1). Furthermore, taxon relative abundances generated with ITS1 were represented consistently over a 10-fold dilution series (1, 1:10, 1:100, 1:1000) mimicking a wide range of sample input concentrations (Figure 1). Notably, the relative abundances of *Blastomyces dermatitidis/gilchristii* was >2-fold underrepresented compared to the expected distribution using all targets.

### ITS1 Mycobiome Recapitulation of Mock Communities

Since virtually any fungal species may cause infection especially in immunocompromised individuals (Czurda and Lion, 2017), the accuracy of the ITS1 mycobiome pipeline was evaluated with a broader range of fungal taxa in two additional mock communities (MC2 and MC3), containing equal mixtures of 20 and 21 fungal species, respectively. Across the 3 mock communities, the ITS1 pipeline correctly detected 51 of 53 species (96.2%), failing only to recover *Sporothrix schenckii* and *Rhizomucor pusillus* most likely due to primer mismatches (Supplementary Table 1). Overrepresentation (>2-fold) occurred with 13.2% of taxa (MC1: 2/20 taxa, MC2: 2/20 taxa, MC3: 4/21 taxa) while 37.7% of taxa were 2-fold underrepresented (MC1: 8/20 taxa, MC2: 5/20 taxa, MC3: 8/21 taxa) compared to the expected relative abundances based on DNA input. Underrepresentation was often associated with mismatches in the primer regions (Supplementary Table 1). Pearson correlation coefficients (PCC) comparing the expected distributions to the mycobiome species relative abundance profiles were *r* = 0.63 (*p* < 0.004) for MC1, *r* = 0.71 (*p* < 0.001) for MC2, and *r* = 0.62 (*p* = 0.002) for MC3.

### Mycobiome Profiling of BAL Specimens During Fungal Pathogenesis

**FIGURE 2:**
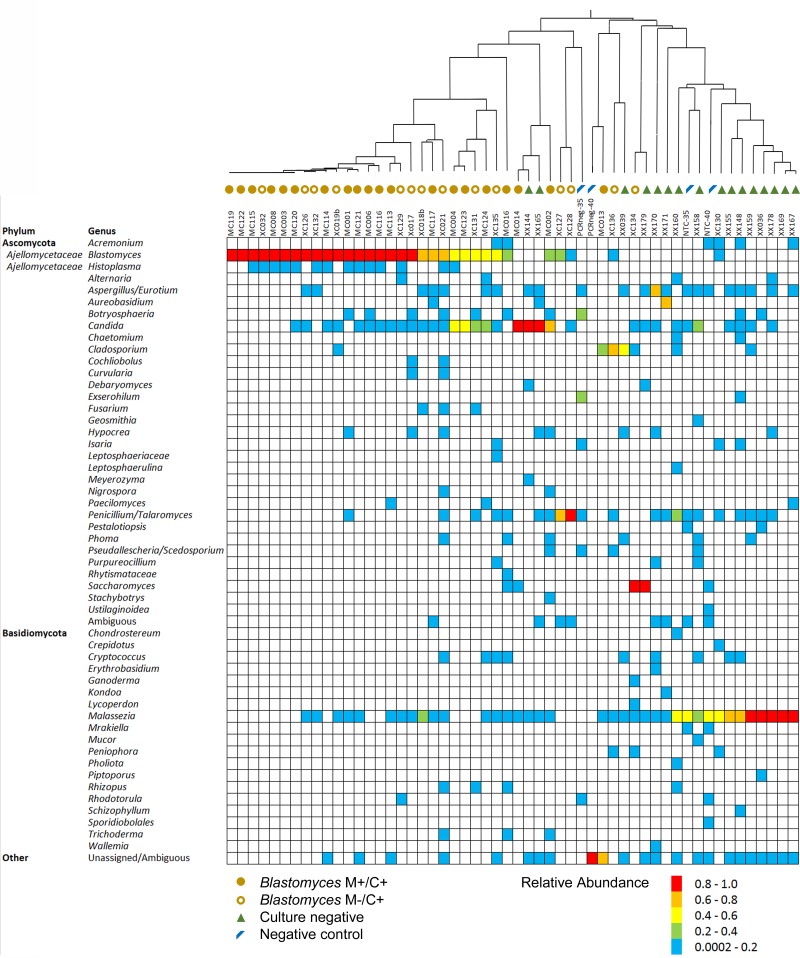
Relative abundance of fungal genera in each of the *Blastomyces* M+/C+ BAL specimens, *Blastomyces* M–/C+ BAL specimens, culture-negative BAL specimens and negative controls. Dendrogram represents UPGMA cluster analysis of Bray–Curtis distances of genus-level summarized OTU abundances.

Since mycobiome analysis using ITS1 provided the most precise recapitulation of MC1, BAL specimens were analyzed using this target to examine the fungal communities in specimens that were culture-positive for *Blastomyces* or culture-negative. Ninety percent (35/39) of *Blastomyces*-culture-positive specimens produced ITS1 amplicons with at least 5000 fungal sequences. In contrast, only 30% (15/50) of culture-negative specimens produced amplicons with at least 5000 fungal reads. Among the 50 BAL specimens with >5000 fungal reads (35 *Blastomyces*-culture-positive, 15 culture-negative), 51 different fungal genera were detected. Distribution of the genera across the dataset was sporadic and sparse with most taxa present in only a few samples (Figure 2). *Blastomyces* was identified in 32 of 35 (91.4%) *Blastomyces*-culture-positive samples but none of the culture-negative or negative control samples. Relative abundance of *Blastomyces* was typically high for both the *Blastomyces* microscopy-positive/culture-positive (M+/C+) (range 0–100%, median 94.6%) and microscopy-negative/ culture-positive (M−/C+) (range 0–100%, median 68.6%) samples (Figure 2) with strong dominance tendencies (percentage of samples where relative abundance of *Blastomyces* was >50%) of 75 and 60%, respectively. Other notable taxa found in both the *Blastomyces*-culture-positive and culture-negative samples included *Candida* present in 64% of samples (relative abundance range 0.1–98.9%), *Aspergillus/Eurotium* present in 38% of samples (relative abundance range 0.1–71.2%), and *Penicillium* present in 38% of samples (relative abundance range 0.1–98.9%). *Malassezia*, present in 70% of samples (relative abundance range 0.1–97.6%), featured prominently with high relative abundances in the culture-negative samples and the NTC negative controls (Figure 2). A UPGMA dendrogram based on Bray–Curtis dissimilarities showed that samples clustered primarily based on these taxa with a separation of the majority of *Blastomyces*-culture-positive samples from most of the culture-negative sample (Figure 2).

**FIGURE 3:**
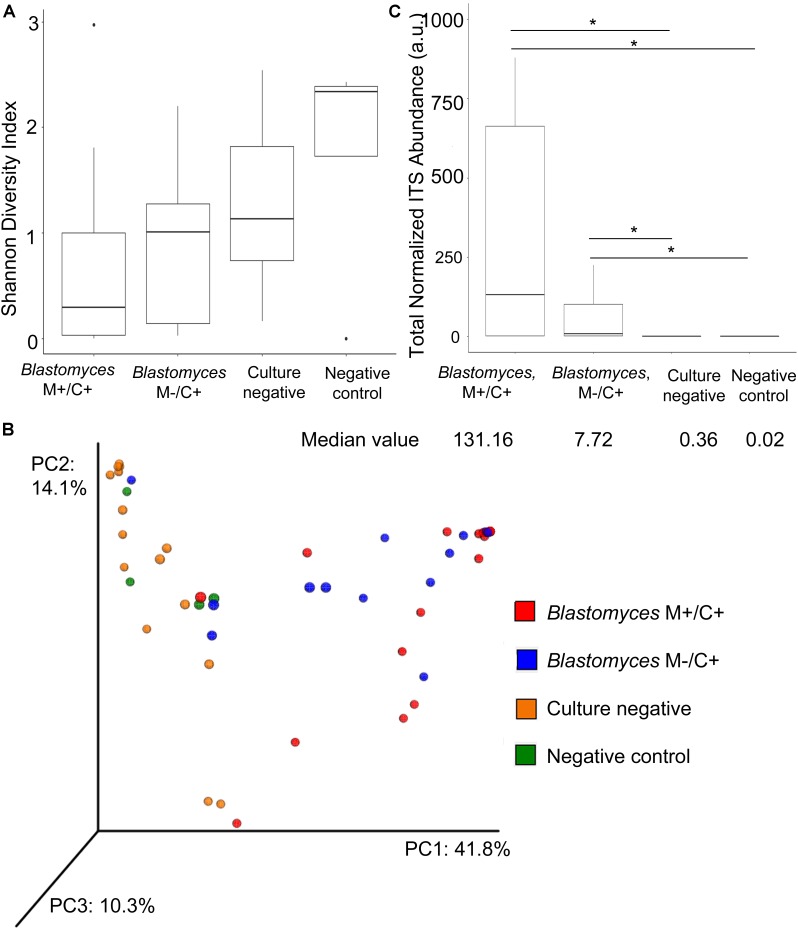
Alpha and beta diversity comparison of fungal communities in *Blastomyces* M+/C+ BAL specimens, *Blastomyces* M–/C+ BAL specimens, culture-negative BAL specimens and negative controls. Boxplots of **(A)** Shannon diversity of genus-level OTU abundance data; there is no statistically significant different between *Blastomyces* M+/C+ samples, *Blastomyces* M–/C+ samples, culture-negative samples, and negative controls (*p* > 0.05). **(B)** PCoA of Bray–Curtis dissimilarities of genus-level summarized OTU abundances. **(C)** Boxplot of total normalized ITS abundances (arbitrary units a.u.). Total normalized ITS abundances of *Blastomyces* M+/C+ samples and *Blastomyces* M–/C+ samples were significantly greater than culture-negative samples and negative controls (^∗^) (*p* < 0.05).

Taxon diversity with respect to abundance and evenness were lower for *Blastomyces* M+/C+ samples compared to *Blastomyces* M−/C+ samples and culture-negative samples as measured by Shannon diversity values based on genus-level summarized OTU abundances. The negative controls exhibited the highest Shannon diversity values, although none of these comparisons were statistically significant (*p* > 0.05) (Figure 3A). PCoA of Bray–Curtis dissimilarities of genus-level summarized OTU abundances showed a clear difference in community composition, distinguishing the *Blastomyces* M+/C+ and *Blastomyces* M−/C+ samples from the culture-negative and negative control samples (Figure 3B). PERMANOVA analysis of genus-level summarized Bray–Curtis dissimilarities confirmed statistically significant differences in the dataset (*F* = 3.83, *R*^2^ = 0.19, *p* = 0.001). Subsequent pairwise *post hoc* analysis showed that the *Blastomyces* M+/C+ samples were statistically different compared to the *Blastomyces* M−/C+ samples, culture-negative samples, and negative control samples (Bonferroni corrected *p* = 0.006, 0.042, 0.036, respectively). None of the other pairwise comparisons were statistically significant (Bonferroni corrected *p* > 0.05) (Supplementary Table 4).

**FIGURE 4:**
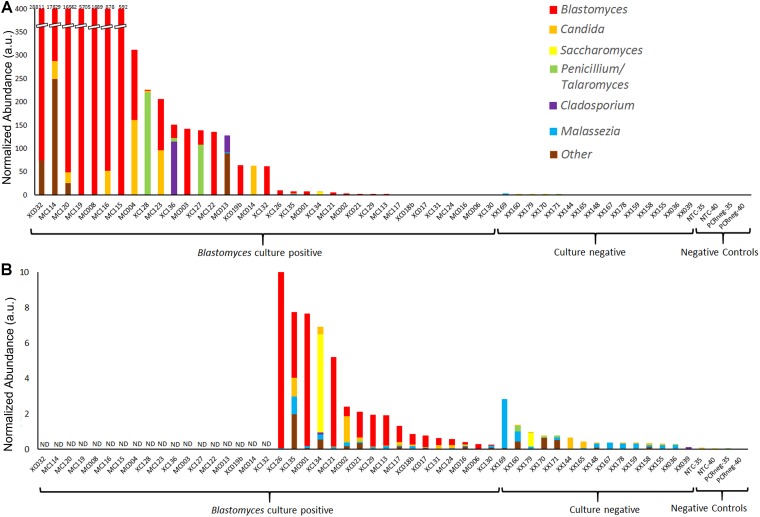
Normalized abundance of fungal taxa detected by ITS1 mycobiome analysis of BAL specimens and negative controls following ITS abundance normalization. Values are expressed as arbitrary units (a.u.) **(A)** Normalized abundance of all *Blastomyces*-culture-positive and culture-negative BAL specimens and negative controls with **(B)** showing an enlarged graph of samples with a total normalized abundance of ≤10 a.u. ND, data not displayed.

The most apparent difference between the *Blastomyces*-culture-positive, culture-negative, and negative control samples was the total quantity of ITS1 amplicon generated; PCR products of *Blastomyces*-culture-positive samples were generated at lower amplification cycle numbers and had higher concentrations than those from culture-negative or negative control samples, reflecting the higher amount of fungal biomass in these samples (Supplementary Table 3). The number of amplification cycles were empirically determined and inversely related to the amount of fungal biomass present in each sample. Therefore using PCR cycle number and PCR product concentration data, as well as the percentage of fungal sequences recovered and the volume of DNA template input into the PCR reaction, an abundance-weighted normalization (Bittinger et al., 2014) was applied to both total and individual taxon relative abundances for each sample (Figure 3C, 4 and Supplementary Table 3). This normalized abundance value was expressed as arbitrary units (a.u.). Total normalized ITS abundance from the *Blastomyces* M+/C+ and M−/C+ samples were significantly greater than culture-negative (*p* = 0.0004, 0.0181, respectively) or negative control samples (*p* = 0.0003, 0.0036, respectively) (Kruskal–Wallis χ^2^ = 27.557, *p* < 0.0001 with Tukey-Kramer *post hoc* pairwise comparisons) (Figure 3C).

Following individual taxon abundance normalization several *Blastomyces*-culture-positive samples, showed abundances of *Candida*, *Saccharomyces*, *Penicillium*/*Talaromyces*, and *Cladosporium* at levels that were substantially higher than those found in the culture-negative samples (Figure 4A,B). Taxon normalized abundances of *Malassezia* were very low across all samples. Interestingly, the mycobiome pipeline failed to detect *Blastomyces* in 3 of the *Blastomyces*-culture-positive samples (MC013, MC014, and XC134). Substantial yeast growth in addition to *Blastomyces dermatitidis/gilchristii* was evident in the cultures of these specimens (Supplementary Table 3) and mycobiome analysis contained high relative abundances of several yeast and other common environmental fungi (*Candida*, *Cladosporium*, *Saccharomyces*), which may have overwhelmed the *Blastomyces* sequences (Figure 2). As well, low relative abundances of *Histoplasma* (<1%) were detected in several *Blastomyces*-culture-positive samples., Although concomitant infection and/or colonization with *Blastomyces* and *Histoplasma* cannot be ruled out, microbiological culture showed no evidence of mixed infections, and this low relative abundance is consistent with the level of cross-talk observed using standard, combinatorial adapters (MacConaill et al., 2018).

Additionally, we analyzed the mycobiome of BAL specimens that were culture-positive for other select fungi. In these samples the cultured fungus was typically the dominant taxon of the mycobiome. Samples culture-positive for *Coccidioides immitis/posadasii* (*n* = 3) and *Scedosporium apiospermum* (*n* = 1) were dominated by these pathogens with relative abundances ranging from 64 to 86% for *C. immitis/posadasii* and 83% for *S. apiospermum* (Supplementary Figure 1). The mycobiome profiles of a set of 2 samples from the same patient yielded *Phaeoacremonium* (*n* = 2) with relative abundances of 83% and 95% the latter of which also contained *Aspergillus* at 3.8% in accordance with the culture results. By contrast, *Histoplasma* was detected in 5 of 6 (83.3%) of samples culture-positive for *H. capsulatum*, however, relative abundances of *Histoplasma* in these samples were low, ranging from 0 to 31% with a median of 2.6% (Supplementary Figure 1); the pathogen never dominated the relative abundance taxon distribution. Predominant taxa in *Histoplasma*-positive samples were *Candida* (*n* = 3), *Pichia* (*n* = 1), *Phoma* (*n* = 1), and *Penicillium* (*n* = 1) (Supplementary Figure 1). Of note, the mock community analysis yielded a relative abundance for *Histoplasma* that was >2-fold underrepresented compared to the expected distribution using ITS1.

## Discussion

In this study, we present an ITS1 amplicon NGS method capable of simultaneously detecting a broad range of potentially clinically significant fungi from mock communities and BAL specimens. Significantly, using this method we observed that the fungal communities in BAL specimens from patients with pulmonary blastomycosis were usually dominated by *Blastomyces*. An ITS1 abundance normalization to account for PCR cycle number and PCR product concentration suggested that fungal biomass was much greater in these samples compared to culture-negative BAL specimens (*p* = 0.0004), which while not unexpected, provides an added level of insight into the data gleaned from the mycobiome analysis. Specifically, taxon-specific abundance normalization demonstrated that several *Blastomyces*-culture-positive samples exhibited the presence of opportunistic yeast and environmental fungi beyond the level seen in the culture-negative samples. The ITS1 mycobiome pipeline also recovered *Coccidioides*, *Phaeoacremonium*, *Aspergillus* and *Scedosporium* from respective culture-positive clinical BAL specimens suggesting its potential as a clinical laboratory diagnostic tool as a direct-from-specimen assay to simultaneously detect and identify a variety of fungal respiratory pathogens without presupposition of their identities.

Although other studies describe methods for generating a mycobiome using ITS amplicon NGS (Bokulich and Mills, 2013; Taylor et al., 2016; De Filippis et al., 2017; Halwachs et al., 2017; Motooka et al., 2017), we offer some improvements to these methods particularly in the area of taxonomic assignment of the OTUs. First, we used a nucleotide BLAST search of the NCBI database to identify any non-fungal OTUs which were subsequently removed from further analysis. Others have also recognized that spurious or non-fungal OTUs can be generated during mycobiome sequencing and recommend eliminating them through a match length-filtering step or *e*-value or bit-score cut-off (Dupuy et al., 2014; Nguyen et al., 2015). Second, taxonomic assignment of OTUs was performed using the FHiTINGS program (Dannemiller et al., 2014) and a database containing ∼118,000 fungal ITS sequences, encompassing ∼13,300 species (Nilsson et al., 2009). Rather than a single match hit, taxonomic classification was assigned by a robust consensus approach derived from congruency among 8 of 10 top hits, thus accounting for ITS sequence similarity between certain taxa. Third, the taxonomies were reviewed to resolve anamorph/teleomorph discrepancies, also recommended by others (Dupuy et al., 2014; Anslan et al., 2018). Because of the dual naming system for fungi, genus-level mycobiome results may yield multiple taxa representing a single species unless the taxonomic classifications are reviewed to harmonize the fungal names. Mycobiome analysis would greatly benefit from the “one fungus = one name” proposal as the ultimate solution to this challenge (Taylor, 2011). For the mock community analysis, *Aspergillus* and *Penicillium* were identified to the section level rather than species level, in keeping with the level of discrimination provided by ITS1 (Skouboe et al., 1999; Geiser et al., 2007; Schoch et al., 2012).

The ITS region is the target of choice for barcoding fungi (Schoch et al., 2012). However, there is some debate over which sub-region is best for mycobiome analysis. As well, there are no true universal primers; all ITS primers discriminate against some fungal taxa (Bellemain et al., 2010; Lindahl et al., 2013; Usyk et al., 2017). Of the target regions and primers tested, we determined that ITS1 most closely reconstructed the expected distributions of MC1, which contained the many of the pathogens of interest. Similarly, Bokulich and Mills (2013) also argue in favor of ITS1 due to shortest mean amplicon lengths, more consistent amplicon lengths between *Ascomycota* and *Basidiomycota*, and higher taxonomic classification accuracy compared to ITS2 or ITS1-2. It is possible that different target regions and optimized primer combinations will be appropriate for different fungal community types (e.g., human vs. environment, or even respiratory vs. gastrointestinal), underscoring the importance of testing pipelines against mock communities consisting of species likely to be recovered from the samples (Nguyen et al., 2015).

We challenged our pipeline with 3 mock communities to cover a broad range of potential fungal lung pathogens. With the exception of *S. schenckii* and *R. pusillus*, which contained mismatches in the primer region, the pipeline recovered the remaining 51 fungal species within the mock communities. However, as noted by many studies on both bacterial 16S rDNA and fungal ITS amplicon-based NGS (Amend et al., 2010; Willner et al., 2012; Bokulich and Mills, 2013; Bowers et al., 2015; Brooks et al., 2015; Fouhy et al., 2016; Taylor et al., 2016; De Filippis et al., 2017), the relative abundances of species recovered by the pipeline differed from the ratio of DNA input to the mock communities. Some species were underrepresented, while others were overrepresented. This skew is not unusual and is due to a combination of variation in rDNA operon copy number, taxon-specific PCR amplification/primer bias, and low Illumina Nextera/MiSeq sequencing depth coverage of regions with high GC content and specific motifs (Amend et al., 2010; Willner et al., 2012; Bokulich and Mills, 2013; Bowers et al., 2015; Brooks et al., 2015; Lan et al., 2015; Fouhy et al., 2016; Taylor et al., 2016; Tyler et al., 2016; Van den Hoecke et al., 2016; Halwachs et al., 2017). Incorrect or imprecise OTU identification may also contribute to mycobiome profile abundance skew, suggesting that further development, expansion and accurate curation of ITS databases may improve fungal community recapitulation accuracy (Usyk et al., 2017). Amplicon-pooled mock communities are typically more representative of species member ratios than DNA-pooled mock communities suggesting that the PCR steps are likely a greater source of bias than the sequencing (Brooks et al., 2015; Fouhy et al., 2016). Despite the over/underrepresentation of certain taxa, the relative abundances of species remained fairly consistent over a broad range of DNA concentrations of MC1, demonstrating the robust sensitivity of the protocol and supporting the validity of inter-sample comparison regardless of differences in DNA input concentration.

Mycobiome analysis of *Blastomyces*-culture-positive and culture-negative BAL specimens revealed a large number and variety of fungal genera present sporadically throughout the dataset. No general respiratory or pathogen-specific “core mycobiome” was deduced amongst these samples. Besides *Blastomyces*, the pathogen of interest, *Candida*, *Aspergillus*/*Eurotium*, and *Talaromyces*/*Penicillium* were present in a substantial number of specimens although the relative abundance values fluctuated dramatically sample-to-sample reflecting a high degree of variability in the lower respiratory tract mycobiome between individuals (Tipton et al., 2017). Other mycobiome studies also report *Candida* spp. and *Aspergillus* spp. as the most frequently detected taxa in the lower respiratory tract of lung transplant, cystic fibrosis, allergic fungal disease, HIV positive, and intensive care unit patients (Bousbia et al., 2012; Delhaes et al., 2012; Cui et al., 2013; Willger et al., 2014; Kim et al., 2015; Kramer et al., 2015; Krause et al., 2016, 2017; Fraczek et al., 2018). The variety of genera detected coupled with the sparse, sporadic distribution may represent a highly variable, fluctuating person-specific fungal lung mycobiome (Cui et al., 2015; Kim et al., 2015; Kramer et al., 2015; Tipton et al., 2017); they may also be transient environmental fungi derived from inhaled air (Kramer et al., 2015), or contamination (Bittinger et al., 2014), and do not represent lung colonization.

PCoA of Bray–Curtis beta-diversity distances suggested a distinction between fungal communities in the *Blastomyces*-culture-positive samples and the culture-negative and negative control samples. However, only the taxon composition of the *Blastomyces* M+/C+ samples was statistically distinct from the 3 other groups: *Blastomyces* M−/C+ samples, culture-negative samples and negative control samples. Accordingly, the relative abundances of *Blastomyces* in the *Blastomyces* M+/C+ samples were higher with fewer alternate taxa resulting in lower (although not significant) alpha diversity than the *Blastomyces* M−/C+ specimens and culture-negative samples. Taxon composition of the *Blastomyces* M−/C+ samples and culture-negative BALs were statistically indistinct with similar alpha diversity indices, likely due to the presence of similar commensal fungi and the propensity for transient and contaminating fungi to be represented in the mycobiome of lower biomass samples such as these (Charlson et al., 2012; Lusk, 2014; Weiss et al., 2014). The presence of alternate fungal taxa may be the cause of the low sensitivity, ∼50% (Arvanitis et al., 2014), of direct microscopic detection of *Blastomyces* in respiratory specimens.

The most prominent feature that distinguished the *Blastomyces*-culture-positive and culture-negative samples was the total ITS abundance normalized to account for the proportion of fungal reads, number of PCR cycles, PCR product concentration and DNA input volume (Bittinger et al., 2014). Total normalized ITS abundance was much higher in the *Blastomyces*-culture-positive BAL samples compared to the culture-negative BALs or negative controls. This was likely the result of greater fungal biomass in the BALs when a pathogen was present than in the absence of a pathogen. Indeed, fungal quantification via qPCR is an acknowledged method of detecting invasive fungal infections in sterile or almost sterile specimens such as BAL, tracheal secretions, tissue from sterile body sites, plasma, serum and whole blood (Czurda and Lion, 2017). As well, 39.2% of BAL samples studied here, predominantly culture-negative samples, failed to generate PCR amplicons or produced less than 5000 mycobiome sequences, presumably due to low fungal biomass. Low fungal biomass and a corresponding difficulty constructing mycobiomes from respiratory samples, particularly those from healthy people, has been noted elsewhere (Charlson et al., 2012).

Absolute quantification of microbial taxon abundances is a useful tool for gleaning additional insights from microbiome surveys (Stammler et al., 2016; Props et al., 2017; Fraczek et al., 2018). Here, we used a taxon abundance-weighted approach (Bittinger et al., 2014) to suggest an abundance of yeast (*Candida*, *Saccharomyces*) and environmental fungi (*Aspergillus*, *Penicillium*/*Talaromyces*, *Cladosporium*) in many of the *Blastomyces* BAL specimens at normalized abundance levels much greater than those found in the culture-negative samples and negative controls. We speculate that these opportunistic fungi proliferated alongside the pathogen *Blastomyces* possibly as a result of lung microbiome perturbation due to blastomycosis and/or immunosuppression. We suggest that for suspected fungal infections by commensal or environmental fungi a taxon abundance-weighted approach may be useful for distinguishing infection from colonization or low-level contamination.

*Malassezia* sp. are common skin commensals (Gaitanis et al., 2012; Findley et al., 2013), and some have suggested that they are also part of the oral or upper respiratory tract mycobiome (Dupuy et al., 2014; Willger et al., 2014; Kim et al., 2015). In this study, *Malassezia* sp. were present in the majority of BAL samples (65.2%), especially the culture-negative samples (100%), at low normalized abundance levels. The genus was also represented in our NTC but not in our PCR negative controls. It may be present in low levels as a lower respiratory tract commensal and/or it may be a pre-PCR-introduced contaminant. Contaminants are problematic in microbiome studies especially with low microbial biomass samples (Lusk, 2014; Salter et al., 2014; Weiss et al., 2014) such as many of the lower respiratory tract samples examined in this study. DNA extraction kits and other laboratory reagents and consumables have been implicated as sources of contamination since they may be sterile but not DNA-free (Salter et al., 2014). Contaminants may also be introduced during specimen collection since conditions and equipment are not pristinely sterile and DNA-free. This finding highlights the importance of sequencing negative controls (Nguyen et al., 2015) and employing methods including the use of appropriate controls to help identify and eliminate contaminants (Willner et al., 2012; Bittinger et al., 2014).

In addition to *Blastomyces*, the ITS1 pipeline detected *Coccidioides*, *Scedosporium*, *Aspergillus*, *Phaeoacremonium*, and *Histoplasma* in BAL specimens in accordance with culture results. With the exception of *Histoplasma*, these pathogens typically exhibited high, nearly exclusive dominance in the mycobiomes of the culture-positive BALs. In contrast, detection of *Histoplasma* was poor with low relative abundance. This could be due to inadequate detection of *Histoplasma* by the ITS1 pipeline, however, it is possible that BALs are not the ideal specimen type for the diagnosis of histoplasmosis. *Histoplasma* has a propensity to migrate to and multiply within the lymphatic system; the respiratory system is a portal of entry but not the ideal niche for growth since the organisms are usually sequestered by the immune system in pulmonary nodules (Wheat et al., 2016). Detection of *Histoplasma* varies depending on the state of disease, which was unknown in this study. Only 10–15% of patient with acute pulmonary histoplamosis have culture-positive sputa, whereas percent positivity increases to 60% with cavitary histoplasmosis (Deepe, 2015). Molecular detection from BALs is likewise challenging with poor sensitivity at 33% reported for a qPCR assay for the detection of *Histoplasma* from culture-positive BALs in contrast to 100% sensitivity from tissue and other respiratory samples (Babady et al., 2011). Presumably this is due to the low concentration of organism in BALs (Babady et al., 2011).

The mycobiome pipeline failed to detect *Blastomyces* and *Histoplasma* in 3 *Blastomyces*-culture-positive BAL specimen and 1 *Histoplasma*-culture-positive BAL specimen, respectively. All four specimens contained an abundance of yeast and other common environmental fungi (*Candida*, *Cladosporium*, *Saccharomyces, Phoma*), suggesting that a technical limitation of the method may occur when quantities of fungal taxa are highly disproportionate. Under these circumstances sequences from the predominate taxa (on) may completely overwhelm sequences from the less-abundant taxon so that it is not detected.

The lack of patient data associated with the BAL specimens is a limitation for this study since we were unable to examine whether the NGS pipeline was able to detect less severe or asymptomatic cases of blastomycosis. As another limitation, the collection and storage parameters of BALs in our study were not controlled. Fecal microbiome studies have demonstrated that sample collection and preservation conditions can affect taxa profiles and relative abundances (Choo et al., 2015; Jenkins et al., 2018). Inconsistent and sub-optimal specimen collection and storage conditions may be a source of bias and false-negative results in this study. Future studies would benefit from defined gold standard methods for BAL collection and storage.

## Conclusion

In conclusion, accurate reconstruction of fungal communities is challenging. The mycobiome analysis described here is capable of detecting a broad range of fungi and useful for describing fungal communities during fungal pathogensis of the lower respiratory tract. We suggest that total and taxon abundance normalizations may help distinguish fungal infection from transient colonization. Precise application of this concept would be aided by future mycobiome investigations of other invasive fungal infections such as aspergillosis. Furthermore, mycobiome analysis shows promise particularly for the clinical microbiology reference laboratory as a “pathogen-agnostic” method (Miller et al., 2013) to detect fungi in cases where traditional microscopy and culture methods yield negative results and yet there remains high index of suspicion of infection.

## Data Availability

Sequences from this project were deposited in the NCBI Sequence Read Archive under accession number PRJNA516455.

## Author Contributions

JK, BC, and LM designed the study. LM and JC performed the experiments and data analysis and interpreted the analyzed results. AS provided bioinformatics analysis scripting support. JK, AS, BC, PW, SH, and DG contributed valuable advice on the analyzed results. LM and JK co-authored the manuscript. All authors have read and approved the final manuscript.

## Conflict of Interest Statement

The authors declare that the research was conducted in the absence of any commercial or financial relationships that could be construed as a potential conflict of interest.
