# Supplementary material for: Mycobiome Sequencing and Analysis Applied to Fungal Community Profiling of the Lower Respiratory Tract During Fungal Pathogenesis
Source: Front Microbiol. 2019 Mar 15;10:512. doi: 10.3389/fmicb.2019.00512 (PMC6428700; doi:10.3389/fmicb.2019.00512)
Supplement: Supplementary file 1 [file Table_1.docx]

Supplementary Material

**Mycobiome Sequencing and Analysis Applied to Fungal Community Profiling of the Lower Respiratory Tract During Fungal Pathogenesis**

**Lisa R. McTaggart^1^, Julia K. Copeland^2^, Anuradha Surendra^3^, Pauline W. Wang^2,4^, Shahid Husain^5,6^, Bryan Coburn^5,6,7^, David S. Guttman^2,4^, Julianne V. Kus^1,7^***

*** Correspondence:** Dr. Julianne Kus: julianne.kus@oahpp.ca

# Supplementary Figures and Tables

**Supplementary Table 1.** Proportional abundance of fungal species in 3 mock communities detected using ITS1 mycobiome analysis. Mock communities were prepared as a mixture of equal quantities (50ng) of genomic DNA of each species. Values in bold represent >2-fold differences from the expected proportional abundances. Primer scores >0 indicate the potential for forward or reverse primer mismatch to negatively affect PCR product generation.

| Phylum | Species | ITS1 Mycobiome Pipeline Result | Expected Proportional Abundance | Mycobiome Proportional Abundance | Forward/Reverse Primer Score |
| --- | --- | --- | --- | --- | --- |
| Mock Community #1  (Total DNA concentration: 2.76 ng/µl) | |  |  |  |  |
| *Ascomycota* | *Aspergillus pseudoglaucus* | *Aspergillus* sec Aspergillus | 5.0% | **2.4%** | 0/0 |
|  | *Aspergillus clavatonanicus* | *Aspergillus* sec Clavati | 5.0% | 7.5% | 0/0 |
|  | *Aspergillus lentulus* | *Aspergillus* sec Fumigati | 5.0% | 4.9% | 0/0 |
|  | *Aspergillus niger* | *Aspergillus* sec Nigri | 5.0% | 7.8% | 0/0 |
|  | *Asperillus terreus* | *Aspergillus* sec Terrei | 5.0% | 6.3% | 0/0 |
|  | *Aspergillus pseudodeflectus* | *Aspergillus* sec Usti | 5.0% | 3.9% | 0/0 |
|  | *Aspergillus versicolor* | *Aspergillus* sec Versicolores | 5.0% | **2.2%** | 0/0 |
|  | *Blastomyces dermatitidis/ gilchristii* | *Blastomyces* *dermatitidis/gilchristii* | 5.0% | **2.2%** | 0/0 |
|  | *Coccidioides immitis/posadasii* | *Coccidioides* *immitis*/*posadasii* | 5.0% | 3.2% | 0/0 |
|  | *Exophiala dermatitidis* | *Exophiala* *dermatitidis* | 5.0% | **1.8%** | 1/1.4 |
|  | *Histoplasma capsulatum* | *Histoplasma capsulatum* | 5.0% | **0.9%** | 0/0 |
|  | *Penicillium chrysogenum* | *Penicillium* sp. | 5.0% | **1.5%** | 0/0 |
|  | *Penicillium corylophilum* | *Penicillium* sec Exilicaulis | 5.0% | 6.8% | 0/0 |
|  | *Purpureocilium lilacinum* | *Purpureocilium* *lilacinum* | 5.0% | **0.3%** | 1/0 |
|  | *Sarocladium strictum* | *Sarocladium* *strictum* | 5.0% | **1.5%** | 1/0 |
|  | *Scedosporium prolificans* | *Scedosporium* *prolificans* | 5.0% | 8.0% | 1/0 |
|  | *Talaromyces columbinus* | *Talaromyces* sec Islandici | 5.0% | 2.5% | 0/0 |
| *Basidiomycota* | *Cryptococcus neoformans* | *Cryptococcus neoformans/gattii neoformans/gattii* | 5.0% | 3.2% | 0/0 |
|  | *Trichosporon asahii* | *Trichosporon asahii* | 5.0% | **15.4%** | 0/0 |
| *Zygomycota* | *Rhizopus oryzae* | *Rhizopus* *oryzae* | 5.0% | **15.2%** | 0/0 |
| Other |  | Other | 0% | 2.4% |  |
|  |  |  |  |  |  |
| Mock Community #2  (Total DNA concentration: 2.60 ng/µl) | |  |  |  |  |
| *Ascomycota* | *Eurotium chevalieri* | *Aspergillus* sec Aspergillus | 5.0% | 3.5% | 0/0 |
|  | *Aspergillus ochraceus* | *Aspergillus* sec Circumdati | 5.0% | **10.5%** | 0/0 |
|  | *Aspergillus clavatus* | *Aspergillus* sec Clavati | 5.0% | **10.8%** | 0/0 |
|  | *Aspergillus flavus* | *Aspergillus* sec Flavi | 5.0% | 8.2% | 0/0 |
|  | *Aspergillus fumigatus* | *Aspergillus* sec Fumigati | 5.0% | 5.4% | 0/0 |
|  | *Emericella nidulans* | *Aspergillus* sec Nidulantes | 5.0% | 4.3% | 0/0 |
|  | *Aspergillus calidoustus* | *Aspergillus* sec Usti | 5.0% | 5.0% | 0/0 |
|  | *Aspergillus sydowii* | *Aspergillus* sec Versicolores | 5.0% | 6.5% | 0/0 |
|  | *Fusarium solani* | *Fusarium/Gibberella* *sp.* | 5.0% | 6.6% | 1/0 |
|  | *Paeciliomyces variotii* | *Paeciliomyces* *variotii* | 5.0% | **2.0%** | 0/0 |
|  | *Rasamsonia argillacea* | *Rasamsonia* *argillacea* | 5.0% | 6.5% | 0/0 |
|  | *Scedosporium apiospermum* | *Scedosporium* *apiospermum* | 5.0% | 7.5% | 1/0 |
|  | *Sporothrix schenckii* | *Sporothrix* *schenckii* | 5.0% | **0.0%** | 1/1.4 |
| *Basidiomycota* | *Cryptococcus gattii* | *Cryptococcus* *neoformans/gattii* | 5.0% | 2.9% | 0/0 |
| *Zygomycota* | *Apophysomyces variabilis* | *Apophysomyces* *variabilis* | 5.0% | 5.0% | 0/0 |
|  | *Cunninghamella elegans* | *Cunninghamella* *elegans* | 5.0% | **0.3%** | 0/0.4 |
|  | *Lichtheimia corymbifera* | *Lichtheimia* *corymbifera* | 5.0% | **1.0%** | 0/0.8 |
|  | *Mucor circinelloides* | *Mucor* *circinelloides* | 5.0% | 9.6% | 0/0 |
|  | *Rhizomucor pusillus* | *Rhizomucor* *pusillus* | 5.0% | **0.0%** | 0/2.2 |
|  | *Rhizopus microsporus* | *Rhizopus* *microsporus* | 5.0% | 2.5% | 0/0 |
| Other |  | Other | 0% | 2.0% |  |
|  |  |  |  |  |  |
| Mock Community #3  (Total DNA concentration: 5ng/µl) | |  |  |  |  |
| *Ascomycota* | *Alternaria alternata* | *Alternaria* *alternata* | 4.76% | 5.2% | 1/0 |
|  | *Aspergillus fumigatus* | *Aspergillus* sec Fumigati | 4.76% | 7.7% | 0/0 |
|  | *Aspergillus flavus* | *Aspergillus* sec Flavi | 4.76% | 7.2% | 0/0 |
|  | *Aspergillus niger* | *Aspergillus* sec Nigri | 4.76% | **9.6%** | 0/0 |
|  | *Aspergillus terreus* | *Aspergillus* sec_Terrei | 4.76% | 5.9% | 0/0 |
|  | *Aspergillus versicolor* | *Aspergillus* sec_Versicolores | 4.76% | 4.7% | 0/0 |
|  | *Candida albicans* | *Candida* *albicans* | 4.76% | 2.7% | 0/0 |
|  | *Candida dubliniensis* | *Candida* *dubliniensis* | 4.76% | **1.3%** | 0/0 |
|  | *Candida glabrata* | *Candida* *glabrata* | 4.76% | **10.5%** | 0.4/0 |
|  | *Candida parapsilosis* | *Candida* *parapsilosis* cplx | 4.76% | **<0.1%** | 0.4/0 |
|  | *Candida tropicalis* | *Candida* *tropicalis* | 4.76% | **1.9%** | 0/0 |
|  | *Cladosporium cladosporioides* | *Cladosporium* sp. | 4.76% | 3.7% | 1/0 |
|  | *Candida lusitaniae* | *Clavispora* *lusitaniae* | 4.76% | **0.4%** | 0/0 |
|  | *Fusarium oxysporum* | *Fusarium*/*Gibberella* sp. | 4.76% | 5.1% | 1/0 |
|  | *Candida guilliermondii* | *Meyerozyma* *guilliermondii* | 4.76% | **2.2%** | 0/0 |
|  | *Penicillium chrysogenum* | *Penicillium* sp. | 4.76% | **2.3%** | 0/0 |
|  | *Candida krusei* | *Pichia* *kudriavzevii* | 4.76% | **11.2%** | 0/0.8 |
|  | *Saccharomyces cerevisiae* | *Saccharomyces* *cerevisiae* | 4.76% | **1.6%** | 0.4/0 |
| *Basidiomycota* | *Sporidiobolus salmonicolor* | *Sporidiobolus* *salmonicolor* | 4.76% | 3.4% | 0/0 |
|  | *Trichosporon asahii* | *Trichosporon* *asahii* | 4.76% | **11.7%** | 0/0 |
| *Zygomycota* | *Mucor racemosus* | *Mucor* *racemosus* | 4.76% | **1.4%** | 0/0 |
| Other |  | Other | 0% | 0.3% |  |

**Supplementary Table 2.** List of primers

| Target | Purpose | Forward primer (5’-3’) | Reverse primer (5’-3’) | Reference |
| --- | --- | --- | --- | --- |
| ITS1-5.8S-ITS2 | Identification | ITS5: GGAAGTAAAAGTCGTAACAAGG | ITS4: TCCTCCGCTTATTGATATGC | White et al., 1990 |
| ITS1 | Mycobiome generation | ITSF: GTAAAAGTCGTAACAAGGTTTC | ITSR: GTTCAAAGAYTCGATGATTCAC | Findley et al., 2013 |
| ITS2 | Mycobiome generation | ITS3: GCATCGATGAAGAACGCAGC | ITS4: TCCTCCGCTTATTGATATGC | White et al., 1990 |
| ITS1-5.8S-ITS2 | Mycobiome generation | ITSF: GTAAAAGTCGTAACAAGGTTTC | ITS4: TCCTCCGCTTATTGATATGC | White et al., 1990; Findley et al., 2013 |
| 18S-28S | Primer binding analysis | ITS-BMB-CR: GTACACACCGCCCGTCG | ITS-LR1: GGTTGGTTTCTTTTCCT | Lane et al., 1985; Vilgalys and Hester 1990 |

White, T.J., Bruns, T., Lee, S., and Taylor, J. (1990). Amplification and direct sequencing of fungal ribosomal RNA Genes for phylogenetics., p 315-322*. In* MA Innis, DH Gelfand, JJ Sninsky and TJ White (ed), PCR protocols: a guide to methods and applications. Academic Press, Cambridge.

Findley, K., Oh, J., Yang, J., Conlan, S., Deming, C., Meyer, J.A., et al. (2013). Topographic diversity of fungal and bacterial communities in human skin. Nature 498,367-370.

Lane, D.J., Pace, B., Olsen, G.J., Stahl, D.A., Sogin, M.L., and Pace, N.R. (1985). Rapid determination of 16S ribosomal RNA sequences for phylogenetic analyses. Proc Natl Acad Sci U S A 82,6955-6959.

Vilgalys, R. and Hester, M. (1990). Rapid genetic identification and mapping of enzymatically amplified ribosomal DNA from several Cryptococcus species. J Bacteriol 172,4238-4246.

**Supplementary Table 3.** Characteristics of *Blastomyces* M+/C+ BAL specimens, *Blastomyces* M-/C+ BAL specimens, BAL specimens culture-positive for other fungi, fungal-culture-negative BAL specimens, and negative controls. Semi-quantitative fungal culture results provide detail on the filamentous fungi and yeast cultured from each sample on IMA and/or BCCG (+++ heavy growth, ++ moderate growth, + little growth). The DNA quantitation criteria (see Materials and Methods) were used to calculate the normalized ITS abundances expressed in arbitrary units (a.u.) for each sample. Samples that failed to generate ITS1 amplicons or <5000 fungal sequence reads are not listed.

| Category | Sample | Culture results | Vol. DNA input (μl) | No. PCR cycles | ITS amplicon conc. (ng/μl) | Fungal read proportion | Total normalized ITS abundance (a.u.) |
| --- | --- | --- | --- | --- | --- | --- | --- |
| *Blastomyces* M+/C+ | | |  |  |  |  |  |
|  | MC001 | *Blastomyces* *dermatitidis/gilchristii* ++ | 4.0 | 35 | 45.5 | 92.5% | 7.66 |
|  | MC002 | *Blastomyces* *dermatitidis/gilchristii* ++ / yeast+++ | 4.0 | 35 | 18 | 72.6% | 2.38 |
|  | MC003 | *Blastomyces* *dermatitidis/gilchristii* +++/ *C. albicans/dubliniensis* + | 4.0 | 30 | 24.4 | 99.9% | 141.94 |
|  | MC004 | Blastomyces *dermatitidis/gilchristii* ++/ *C. glabrata* ++ | 4.0 | 30 | 53.5 | 99.9% | 311.20 |
|  | MC006 | *Blastomyces dermatitidis/gilchristii +++* | 9.5 | 35 | 8.13 | 44.1% | 0.27 |
|  | MC008 | *Blastomyces dermatitidis/gilchristii +++* | 1.0 | 28 | 20.3 | 99.9% | 1889.35 |
|  | MC013 | *Blastomyces* *dermatitidis/gilchristii* +/ yeast +++ | 4.0 | 30 | 21.9 | 99.7% | 127.11 |
|  | MC014 | *Blastomyces* *dermatitidis/gilchristii* +/ yeast+++ | 4.0 | 30 | 10.7 | 99.8% | 62.13 |
|  | MC016 | *Blastomyces dermatitidis/gilchristii ++* | 9.5 | 35 | 6.06 | 81.9% | 0.38 |
|  | MC113 | Blastomyces *dermatitidis/gilchristii* ++/ yeast + | 4.0 | 35 | 11.1 | 94.6% | 1.91 |
|  | MC114 | *Blastomyces dermatitidis/gilchristii* +++ | 1.0 | 25 | 28.8 | 83.1% | 17829.20 |
|  | MC115 | *Blastomyces* *dermatitidis/gilchristii* +++/ yeast + | 1.0 | 28 | 6.44 | 98.7% | 592.02 |
|  | MC116 | *Blastomyces* *dermatitidis/gilchristii* +++/ yeast+ | 1.0 | 28 | 9.67 | 97.5% | 877.87 |
|  | MC117 | *Blastomyces* *dermatitidis/gilchristii* ++/ yeast + | 4.0 | 35 | 7.66 | 94.3% | 1.31 |
|  | MC119 | *Blastomyces dermatitidis/gilchristii +* | 1.0 | 25 | 8.85 | 86.5% | 5704.89 |
|  | MC120 | *Blastomyces* *dermatitidis/gilchristii* ++ / yeast++ | 1.0 | 25 | 23.1 | 96.2% | 16562.03 |
|  | MC121 | *Blastomyces dermatitidis/gilchristii +++* | 4.0 | 33 | 7.61 | 93.9% | 5.20 |
|  | MC122 | *Blastomyces dermatitidis/gilchristii +++* | 2.0 | 30 | 12.7 | 91.4% | 135.21 |
|  | MC123 | *Blastomyces dermatitidis/gilchristii* ++/ yeast ++ | 2.0 | 30 | 17.8 | 99.5% | 206.24 |
|  | MC124 | *Blastomyces dermatitidis/gilchristii* ++/ yeast+ | 4.0 | 35 | 4.17 | 74.2% | 0.56 |
| *Blastomyces* M-/C+ | | |  |  |  |  |  |
|  | XC017 | *Blastomyces dermatitidis/gilchristii ++* | 4.0 | 35 | 4.97 | 83.9% | 0.76 |
|  | XC018b | *Blastomyces dermatitidis/gilchristii +* | 9.5 | 35 | 13.9 | 80.3% | 0.85 |
|  | XC019b | *Blastomyces dermatitidis/gilchristii +* | 2.0 | 30 | 7.04 | 77.9% | 63.87 |
|  | XC021 | *Blastomyces dermatitidis/gilchristii ++* | 4.0 | 35 | 12.5 | 92.8% | 2.11 |
|  | XC032 | *Blastomyces dermatitidis/gilchristii ++* | 1.0 | 25 | 38.7 | 99.9% | 28810.86 |
|  | XC126 | *Blastomyces dermatitidis/gilchristii +* | 4.0 | 33 | 14.5 | 94.6% | 9.98 |
|  | XC127 | *Blastomyces dermatitidis/gilchristii* ++/ yeast + | 2.0 | 30 | 12.1 | 98.7% | 139.01 |
|  | XC128 | *Blastomyces dermatitidis/gilchristii* +/ yeast +/bacteria +++ | 2.0 | 30 | 19.3 | 99.9% | 224.39 |
|  | XC129 | *Blastomyces* *dermatitidis/gilchristii* ++ / yeast + | 4.0 | 35 | 14 | 76.3% | 1.94 |
|  | XC131 | *Blastomyces dermatitidis/gilchristii +* | 9.5 | 35 | 8.02 | 99.6% | 0.61 |
|  | XC132 | *Blastomyces dermatitidis/gilchristii +* | 2.0 | 30 | 5.58 | 95.5% | 62.05 |
|  | XC130 | *Blastomyces dermatitidis/gilchristii ++* | 9.5 | 35 | 3.1 | 93.1% | 0.22 |
|  | XC134 | *Blastomyces dermatitidis/gilchristii* +/ yeast +++ | 4.0 | 33 | 11.1 | 85.6% | 6.91 |
|  | XC135 | *Blastomyces dermatitidis/gilchristii* +/ yeast +/ bacteria +++ | 4.0 | 33 | 14.8 | 71.7% | 7.72 |
|  | XC136 | *Blastomyces dermatitidis/gilchristii +* | 2.0 | 30 | 13 | 99.7% | 150.83 |
| Other fungi, culture-positive | | |  |  |  |  |  |
|  | MC009 | *Histoplasma capsulatum* ++/ yeast ++ | 9.5 | 35 | 10.8 | 50.6% | 0.42 |
|  | XC025 | *Histoplasma capsulatum ++* | 1.0 | 28 | 10.8 | 99.6% | 1002.01 |
|  | XC026 | *Histoplasma capsulatum ++* | 4.0 | 35 | 9.56 | 52.7% | 0.92 |
|  | XC027 | *Histoplasma* *capsulatum* + / yeast + | 4.0 | 30 | 40 | 99.9% | 232.66 |
|  | XC028 | *Histoplasma* *capsulatum* +++ / yeast +++ | 4.0 | 30 | 25.7 | 98.9% | 147.90 |
|  | XC029 | *Histoplasma* *capsulatum* +/ yeast + | 4.0 | 35 | 5.26 | 59.9% | 0.57 |
|  | XC023 | *Coccidioides immitis/posadasii* + | 4.0 | 35 | 13 | 73.6% | 1.74 |
|  | XC024 | *Coccidioides* *immitis/posadasii* ++ | 9.5 | 35 | 3.64 | 99.6% | 0.28 |
|  | XC030 | *Coccidioides immitis/posadasii* ++ / Basidiomycota-like sp. + | 4.0 | 35 | 27.1 | 73.5% | 3.62 |
|  | MC010 | *Phaeoacremonium sp. +++* | 4.0 | 30 | 12.3 | 54.2% | 38.79 |
|  | MC011 | *Aspergillus fumigatus ++/ Phaeoacremonium sp. +* | 4.0 | 30 | 28.4 | 98.6% | 163.02 |
|  | XC031 | *Scedosporium apiospermum +* | 4.0 | 35 | 8.71 | 25.2% | 0.40 |
| Culture-negative | |  |  |  |  |  |  |
|  | XX036 | bacteria + | 9.5 | 35 | 3.8 | 85.6% | 0.25 |
|  | XX039 | - | 9.5 | 35 | 2.32 | 59.5% | 0.11 |
|  | XX144 | - | 9.5 | 35 | 10.4 | 80.0% | 0.64 |
|  | XX148 | - | 9.5 | 35 | 5.28 | 89.2% | 0.36 |
|  | XX155 | - | 9.5 | 35 | 5.07 | 76.4% | 0.30 |
|  | XX158 | - | 9.5 | 35 | 4.26 | 97.5% | 0.32 |
|  | XX159 | - | 9.5 | 35 | 4.86 | 90.4% | 0.34 |
|  | XX160 | - | 4.0 | 35 | 12.3 | 61.2% | 1.37 |
|  | XX167 | - | 9.5 | 35 | 4.97 | 94.6% | 0.36 |
|  | XX169 | - | 4.0 | 35 | 19.3 | 80.1% | 2.81 |
|  | XX165 | - | 9.5 | 35 | 6.06 | 87.4% | 0.41 |
|  | XX170 | - | 4.0 | 35 | 4.75 | 88.3% | 0.76 |
|  | XX171 | - | 4.0 | 35 | 7.2 | 57.8% | 0.76 |
|  | XX178 | - | 9.5 | 35 | 4.86 | 91.6% | 0.34 |
|  | XX179 | - | 4.0 | 35 | 7.88 | 67.5% | 0.97 |
| Negative controls | |  |  |  |  |  |  |
|  | NTC-35 | NTC | 9.5 | 35 | 1 | 72.6% | 0.06 |
|  | NTC-40 | NTC | 9.5 | 40 | 13 | 98.1% | 0.03 |
|  | PCRneg-35 | PCR negative | 9.5 | 35 | 0.1 | 99.8% | 0.01 |
|  | PCRneg-40 | PCR negative | 9.5 | 40 | 2 | 16.7% | 0.0008 |

**Supplementary Table 4.** PERMANOVA of Bray-Curtis dissimilarities between fungal communities represented by genus-level OTU abundances in *Blastomyces* M+/C+ BAL specimens, *Blastomyces* M-/C+ BAL specimens, culture-negative BAL specimens and negative controls. *denotes statistical significance.

|  | df | SS | MS | F | R^2^ | P |  |
| --- | --- | --- | --- | --- | --- | --- | --- |
| Category | 3 | 3.1748 | 1.05828 | 3.8276 | 0.1868 | 0.001* |  |
| Residuals | 50 | 13.8244 | 0.27649 |  | 0.8132 |  |  |
| Total | 53 | 16.9992 |  |  | 1.0000 |  |  |
| Post-hoc |  |  |  | F | R^2^ | p | p-adj. |
| *Blastomyces* M+/C+ vs. *Blastomyces* M-/C+ | | | | 26.132 | 0.442 | 0.001 | 0.006* |
| *Blastomyces* M+/C+ vs. Culture-negative | | | | 8.306 | 0.201 | 0.007 | 0.042* |
| *Blastomyces* M+/C+ vs. Negative controls | | | | 10.069 | 0.314 | 0.006 | 0.036* |
| *Blastomyces* M-/C+ vs. Culture-negative | | | | 2.199 | 0.073 | 0.123 | 0.738 |
| *Blastomyces* M-/C+ vs. Negative controls | | | | 0.270 | 0.016 | 0.810 | 1.000 |
| Culture-negative vs. Negative controls | | | | 0.850 | 0.048 | 0.388 | 1.000 |

**Supplementary Figure 1. Relative abundance of fungal genera in BAL specimens that were culture positive for fungi other than *Blastomyces*.**
